# Preferential transmission of minority and drug-resistant clones in polyclonal infections in Mali

**DOI:** 10.1186/s12936-025-05298-6

**Published:** 2025-04-05

**Authors:** Leen N. Vanheer, Emilia Manko, Almahamoudou Mahamar, Jody Phelan, Koualy Sanogo, Youssouf Sinaba, Sidi M. Niambele, Adama Sacko, Sekouba Keita, Ahamadou Youssouf, Makonon Diallo, Harouna M. Soumare, Kjerstin Lanke, Djibrilla Issiaka, Halimatou Diawara, Sekou F. Traore, Lynn Grignard, Alassane Dicko, Chris Drakeley, Susana Campino, William Stone

**Affiliations:** 1 Department of Infection Biology, Faculty of Infectious and Tropical Diseases, London School of Hygiene and Tropical Medicine, London, UK; 2 Malaria Research and Training Centre, Faculty of Pharmacy and Faculty of Medicine and Dentistry, University of Sciences Techniques and Technologies of Bamako, Bamako, Mali; 3 Department of Medical Microbiology and Radboud Center for Infectious Diseases, Radboud University Medical Center, Nijmegen, The Netherlands

**Keywords:** Malaria, *Plasmodium falciparum*, Gametocytes, Transmission, Comparative genomics, Genetic diversity, Artemisinin-combination therapy, Drug resistance

## Abstract

**Background:**

In polyclonal human malaria infections, the roles of individual clones in human-to-mosquito transmission and their relative transmissibility remain poorly understood. In addition, mutations conferring drug resistance can result in a transmission advantage or disadvantage.

**Methods:**

Amplicon sequencing of complexity of infection and drug resistance markers was used to analyse post-treatment stage-specific malaria parasite dynamics in human blood infections and in the midguts of mosquitoes that became infected after direct membrane feeding assays (DMFAs). Blood samples originated from 50 asymptomatic *Plasmodium falciparum* gametocyte-carrying participants. These were collected prior to treatment and at five timepoints over 28 days following a three-day artemisinin-based combination therapy (ACT) regimen of dihydroartemisinin-piperaquine or pyronaridine-artesunate at the Ouélessébougou Clinical Research Unit of the Malaria Research and Training Centre of the University of Bamako (Bamako, Mali). At each study visit, DMFAs were conducted.

**Results:**

A total of 57 *Pfcsp* haplotypes and 53 *Pftrap* haplotypes were identified, indicating high genetic diversity among parasite clones. Prior to treatment, human infections were more often polyclonal and had a higher median multiplicity of infection (MOI; 3 (IQR 2–5)), compared to mosquito midgut infections (1 (IQR 1–2)). At this timepoint, it is likely that some clones detected in human blood are not producing gametocytes and are, therefore, not contributing to mosquito transmission. Minority clones preferentially transmitted, and these same clones often persisted in the human blood samples post-treatment. These observations mirror the rapid decline in asexual parasite density that occurs after ACT initiation, and the more persistent circulation of gametocytes. The data, therefore, suggests that asexual gametocyte-non-producing clones outnumber the gametocyte-producing clones at baseline, yet it is these gametocyte-producing minority clones that are transmitted to and surviving in mosquitoes. Certain haplotypes were also found to be more prevalent in human samples compared to mosquito infections, and vice versa, with 12.6% of haplotypes at baseline exclusively observed in mosquitoes. Along with this, varying odds of transmission for different parasite clones were observed, indicating that there are inherent clonal differences in gametocyte productivity or viability. To assess the transmission of drug-resistant clones, the overall prevalence of molecular markers of drug resistance was determined in both human and mosquito hosts, followed by a pairwise comparison between human blood infections and paired infected midguts. This showed that Asn51Ile in *Pfdhfr* and Lys540Glu in *Pfdhps* may have a transmission advantage under ACT, while Ala613Ser in *Pfdhps* may confer a transmission disadvantage.

**Conclusions:**

Overall, these findings indicate that parasite dynamics and clonal transmissibility are highly complex, even after ACT. This complexity may have important epidemiological implications, as it suggests the transmission of minority clones and highlights the impact of drug resistance markers on transmissibility.

**Supplementary Information:**

The online version contains supplementary material available at 10.1186/s12936-025-05298-6.

## Background

The cycle of *Plasmodium* transmission between human and mosquito hosts relies on the parasite’s ability to produce gametocytes. These gametocytes, when ingested by a mosquito, transform into gametes, which undergo sexual recombination, then develop into zygotes/ookinetes, which cross the epithelial wall of the mosquito midgut to develop into oocysts. Various factors such as gametocyte viability and density, host immune responses, and the complexity of the infection can influence this process [1–5].

In regions where *Plasmodium falciparum* infections are endemic, individuals are often infected by more than one parasite strain at the time, leading to polyclonal infections, which may be the result of clonal co-transmission (a single mosquito infection with multiple clones) or serial superinfections (multiple mosquito infections, each with a single clone or multiple clones). This multiplicity of infection (MOI), indicating the number of clones within an infection, and the complexity of infection (COI), referring to specific genetic characteristics of these clones, are important for understanding *Plasmodium* epidemiological patterns and transmission dynamics. In regions with high malaria transmission and especially among asymptomatic individuals, infections usually exhibit greater multiclonality, and the various parasite clones compete with one another for replication and transmission, though the resources they compete for are not well understood [6–8]. Although most parasite stages are haploid, the zygote formed after fertilization in the mosquito midgut is diploid. At this stage, new parasite haplotypes emerge when genetically distinct gametocytes are transmitted to mosquitoes, where recombination occurs during sexual reproduction, increasing genetic diversity in subsequent generations.

In polyclonal human infections, the contribution of distinct clones to transmission and their relative transmissibility have largely remained unexplored, as have the parasite genetic factors that may influence this. In addition, due to the parasite’s complex life cycle, many different population bottlenecks and selective pressures are encountered throughout human host and organ transitions, which may intensify random genetic drift and natural selection [9].

Transmission represents a major population bottleneck in the parasite life cycle, as natural mosquito infections typically harbour only about 2–5 oocysts, in contrast to the approximately 10^11^ asexual parasites found in an infected host [10]. In a prior study of transmission using naturally-infected paired human and mosquito samples, haplotype diversity was found to be greater in mosquitoes than in humans, establishing the mosquito vector as a reservoir of genetic diversity in the malaria parasite populations [8]. This finding is seemingly contradictory to observations that not all clones present in the human blood transmit to mosquitoes. This could indicate a new infection which may not have had time yet to generate mature gametocytes, or a potential failure to produce gametocytes altogether [8, 11]. On the other hand, transmission of clones that were undetectable in the blood stream was reported in multiple studies [1, 11, 12], and it is commonly observed that not all individuals with confirmed gametocytes are able to infect mosquitoes [13–15]. Such observations may be more likely to reflect the density dependence of successful transmission, the technical limitations in detecting low density clones, or the activity of human or mosquito immune transmission blockage, rather than genetic unviability.

Artemisinin-based combination therapy (ACT) is the current first-line treatment for uncomplicated *P. falciparum* malaria, however, its gametocytocidal and transmission-blocking effects vary widely. After treatment with commonly used artemisinin-based combinations, such as dihydroartemisinin-piperaquine (DHA-PPQ), pyronaridine-artesunate (PY-AS) or artesunate-amodiaquine, gametocytes and transmission can persist up to 28 days after treatment initiation [13, 16]. A recent study found that post-treatment parasite dynamics of blood stage parasites are highly complex despite efficacious treatment [17]. Post-treatment parasite dynamics in both human blood and cognate infected mosquito midguts remain unstudied.

The rise of anti-malarial drug resistance has driven the adoption of new therapeutic approaches. Investigating the effect of drug resistance on transmissibility is crucial, as any transmission advantages conferred by resistance-linked mutations could expedite the spread of these dangerous parasite strains and compound a developing public health crisis. Studies have shown evidence of increased gametocytaemia and increased transmission in infections with chloroquine (CQ) resistant *P. falciparum* parasites, compared to infections with parasites sensitive to this anti-malarial [18]. For sulfadoxine-pyrimethamine (SP), it has been found that resistant parasites produce more gametocytes, but their transmission potential remains unclear [19, 20]. In addition, host-specific selection of drug resistant polymorphisms has been observed, reporting contrasting single nucleotide polymorphisms (SNPs) in the *Pfdhfr* gene in human and mosquito hosts [21].

This study characterises parasite clones and investigates the clonal transmissibility of 50 naturally-infected asymptomatic *P. falciparum* gametocyte carriers at different timepoints following a course of ACT (dihydroartemisinin-piperaquine or pyronaridine-artesunate) treatment in South-Western Mali, a region of intense malaria transmission, where high SP resistance and persistent chloroquine resistance have been reported [22, 23]. Gametocyte carriage was an essential recruitment criterion, allowing for inference of parasite stage identity based on the differential clearance times of sexual and asexual stage parasites after ACT. In addition, the relatedness of molecular markers of anti-malarial drug resistance and transmission potential is investigated.

## Methods

### Study site, sample collection and feeding assays

Human blood samples of 50 individuals with microscopy detectable *P. falciparum* gametocytes (≥ 1 gametocyte against 500 white blood cells on thick smear, equating to ≥ 16 gametocytes per μL of blood with an assumed WBC density of 8000/μL blood), and mosquitoes infected by direct membrane feeding assay (DMFA) were obtained from a clinical trial performed in Mali in 2019 [13] (Supplementary Fig. 1). Individuals were treated with a full 3-day course of dihydroartemisinin-piperaquine or pyronaridine-artesunate and were followed up until 49 days after treatment initiation. Re-treatment with dihydroartemisinin-piperaquine was administered at day 21 to prevent re-infection. Blood samples were taken at each study visit for parasite density measurements and mosquito feeding assays. Blood samples for molecular analyses were stored in RNA protect cell reagent (Qiagen, Hilden, Germany) and frozen at − 70 °C until nucleic acid extraction. At each study visit, 75 insectary-reared *Anopheles gambiae *sensu lato* (s.l.)* mosquitoes were allowed to feed for 15–20 min on whole blood collected from participants. Surviving mosquitoes were dissected 7 days after feeding to allow for parasite establishment and oocyst development. Midguts were stained with 1% mercurochrome solution and the number of oocysts in the lamina of the midguts was recorded by trained technicians, after which midguts were stored in RNA protect cell reagent at − 80 °C until extraction. Permission to conduct this study was obtained from the London School of Hygiene and Tropical medicine Research Ethics Committee (reference number 17507) and the University of Sciences Techniques and Technologies of Bamako Ethical Committee (reference number 2019/67/ CE/FMPOS) and performed in accordance with relevant guidelines and regulations. The trial was registered on ClinicalTrials.gov (NCT04049916). Written informed consent was obtained from all subjects and/or their legal guardians prior to sample collection. For minor participants (5–17 years old), informed consent for study participation was obtained from their parent and/or legal guardian.

### Nucleic acid extraction and parasite quantification

DNA was extracted from 83.3 μL whole blood using a MagNAPure LC automated extractor (Total Nucleic Acid Isolation Kit High Performance; Roche Applied Science, Indianapolis, IN, USA). Ring stage parasitaemia was determined by reverse-transcriptase quantitative PCR (RT-qPCR) targeting skeleton-binding protein 1 (SBP1) [24]. Female and male gametocytes were quantified by RT-qPCR targeting *PfCCP4* and *PfMGET,* respectively, as previously described [25], before, during and after treatment in both treatment groups (dihydroartemisinin-piperaquine and pyronaridine artesunate). Before treatment initiation, molecular assessment of asexual parasites was conducted in 48 out of 50 individuals. For each time point in the infectivity assay, if an individual had up to three infected mosquitoes, all were selected. If there were more than three infected mosquitoes that resulted from the infectivity assay at a certain time point for a certain individual, a random selection of three infected mosquitoes was made (Supplementary Fig. 1) and oocyst DNA was extracted using the Qiagen DNeasy blood & tissue kit with overnight proteinase K lysis and eluted in 50 µL EB.

### Genotyping of human blood samples and infected mosquito midguts

**Fig. 1:**
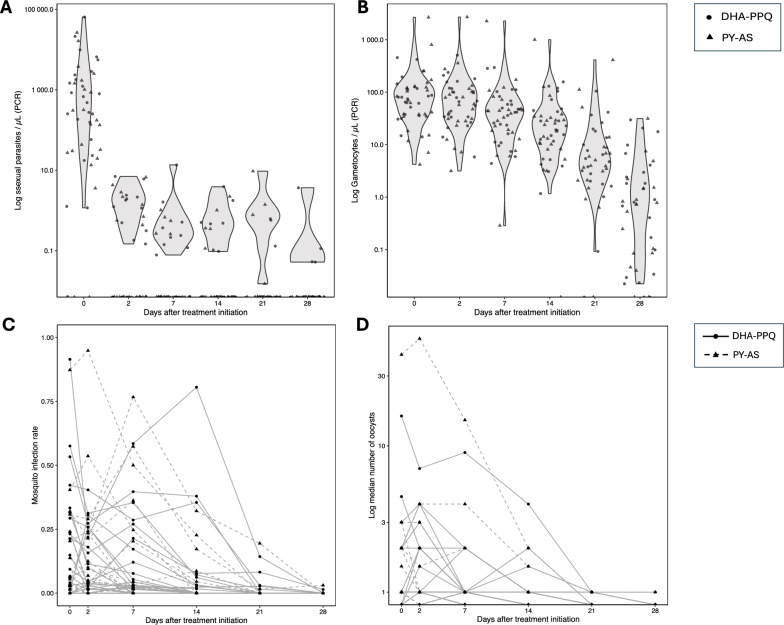
Infection dynamics. Violin plots showing **A** Asexual parasite densities (parasites / μL) and **B** gametocyte densities measured by qPCR (gametocytes / μL) at each study visit. Each point represents a study participant, with circles indicating participants in the dihydroartemisinin-piperaquine group and triangles indicating participants in the pyronaridine-artesunate group. Line graphs represent **C** mosquito infection rate and **D** median number of oocysts in infected mosquito midguts. Each line represents one individual, with full lines and circles marking individuals in the dihydroartemisinin-piperaquine group and dashed lines and triangles indicating participants in the pyronaridine-artesunate group. The median number of oocysts in D was set to zero if no mosquitoes were infected at a certain timepoint for a certain participant. *DHA-PPQ*  dihydroartemisinin-piperaquine, *PY-AS*  pyronaridine-artesunate

Amplicon sequencing to determine complexity of infection was performed as previously described, targeting the circumsporozoite protein (*Pfcsp;* nucleotides 1506–1794) and the thrombosporin-related anonymous protein (*Pftrap*, nucleotides 1242–1562) [26–28] (Supplementary Table 1). A total of 195 human blood samples from 50 trial participants and 315 mosquito midgut samples from DMFAs conducted on these participants were selected for genotyping. Briefly, an approximate 300 base pairs (bp) region of each gene was amplified by multiplexed PCR in duplicates and an in-line barcode was added to the primer sequences, allowing pooling of amplicons. Sequencing of amplicon pools was then performed using overlapping 250 bp paired-end MiSeq Illumina reads at Genewiz (Azenta Life Sciences). In vitro* P. falciparum* culture DNA of parasite lines 3D7 and HB3 was used to assess the limit of detection of the assay (Supplementary Fig. 2). Amplicon sequencing of *P. falciparum* anti-malarial resistance markers using nanopore sequencing was performed as previously described [29], on 50 human and 87 cognate mosquito specimens which fed on blood samples from 35 of these individuals, sampled on day 2 after treatment initiation (Supplementary Fig. 1). This timepoint was chosen since nearly all asexual parasites were removed from circulation by ACT within 48 h of treatment commencement, while gametocyte densities were still high (Fig. 1). Primer sequences and multiplexed PCR conditions for amplification of the *Pfcrt*, *Pfdhfr*, *Pfdhps*, *Pfmdr1* and *Pfkelch13* genes can be found in Supplementary Table 2. Library preparation was carried out according to manufacturer instructions using ONT kit SQK-NBD114.96 following the ‘ligation sequencing amplicons-native barcoding’ protocol.

### Bioinformatics and statistical analysis

The haplotypeR package was used to determine complexity of infection per sample [30], with minor modifications to extend certain functions (https://github.com/leenvh/haplotypR_funs) and using the parameters minMMrate 0.5, minOccGen 2, minCov 3, detectionLimit 1/200, minOccHap 2, MinCovSample 20. Haplotypes were only considered as real if they were present in both technical replicates, thereby minimising the risk of detecting haplotypes caused by amplification or sequencing errors. Correlations between markers and replicates were assessed by Spearman’s rank correlation coefficient. Haplotype networks were constructed using the R package *Pegas* [31]. Base calling of nanopore sequencing data was performed using an in-house pipeline, utilizing *Guppy* (Version 6.5.7). Further bioinformatics analysis was done by in-house demultiplexing script (https://github.com/LSHTMPathogenSeqLab/amplicon-seq/tree/main) and drug resistant polymorphisms were analysed by the malaria profiler tool [32]. Frequencies of molecular markers of drug resistance were compared between human and mosquito populations by Fisher exact test. Visualizations and statistical analyses were performed in R (version 4.3.2) and can be found on https://github.com/leenvh/Amplicons-falciparum-MOI. The sequence data presented in this study can be found in the European Nucleotide Archives (ENA, Project accession PRJEB73503).

## Results

### Stage-specific infection dynamics and infectivity before and after treatment

Before treatment initiation, asexual parasites were detectable by RT-qPCR in 91.6% (44/48) of individuals (median density 396.77 parasites/μL, IQR 54.15–1931.89). All individuals were recruited based on the presence of gametocytes by microscopy and gametocytes were detectable by RT-qPCR in all participants at baseline (median density 77.33 gametocytes/μL, IQR 37.1–124.02). A total of 66% (33/50) of study participants were able to infect mosquitoes at baseline, with a median infection rate of 14.9% in mosquitoes (IQR 3.51–31.4). At 48 h after treatment initiation, nearly all asexual parasites were cleared in both treatment groups (only densities of ≤ 7 asexual parasites/μL remained in 20 individuals, median 1.59, IQR 0.55–2.5), while gametocytes densities showed a slow decline (Fig. 1A-B). Parasite prevalences and densities were comparable between treatment groups during follow-up (Supplementary Table 3, Fig. 1A-B). Mosquito infection rates declined after treatment, but transmission persisted until day 28 in some individuals in both groups. The median oocyst density in infected mosquitoes at baseline was 1 (IQR 1–2) (Fig. 1C-D, Supplementary Table 4).

### Majority of infections in asymptomatic gametocyte carriers are polyclonal and highly diverse

**Fig. 2:**
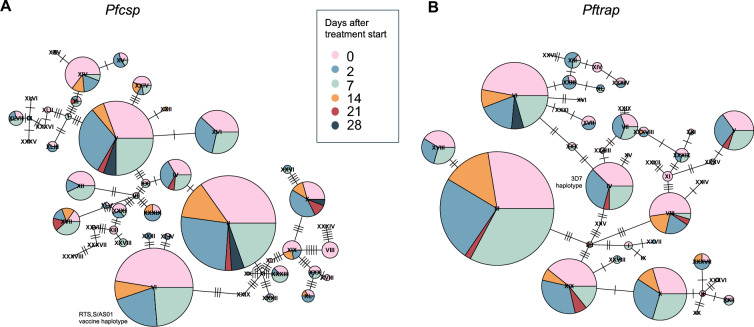
Haplotype network showing genetic diversity. Haplotype or minimal-spanning network constructed using **A** *Pfcsp* and **B** *Pftrap.* Each node represents a haplotype, each segment within the node represents a study timepoint, and is proportionally sized to the number of sequences present in the segment and node. The number of ticks between nodes represents the number of genetic differences between nodes

To investigate the *P. falciparum* genotypes in human and mosquito samples, human blood samples and infected mosquito midgut samples from DMFAs conducted on these participants were genotyped. Amplicons for *Pfcsp* and *Pftrap* were successfully sequenced (median coverage of 1002 and 944, respectively) for 168 (86.2%) human samples and 151 (47.9%) mosquito samples (Supplementary Table 5). Multiplicity of infection was highly correlated between technical replicates of the same sample (*Pfcsp* marker spearman correlation = 0.76, p < 0.0001; *Pftrap* marker spearman correlation = 0.75, p < 0.0001) and between the two sequenced markers (spearman correlation = 0.8, p < 0.0001; Supplementary Fig. 3). A total of 1,220 amplicons across both markers were analysed, identifying a total of 57 *Pfcsp* haplotypes and 53 *Pftrap* haplotypes, reflecting 28 and 20 positions with single nucleotide polymorphisms (SNPs), respectively (Fig. 2).

Pre-treatment infected human blood samples were successfully genotyped for complexity of infection markers for 43 out of 50 potential study participants. Of these, 88.4% (38/43) of infections were polyclonal, with a median MOI of 3 (IQR 2–5). Clonality in mosquito infections, incubated for 7 days post-feeding to allow for oocyst development, was lower than in matched blood stage parasites, with only 36.5% (19/52) of midguts being polyclonal and a median MOI of 1 (IQR 1–2). At days 2 and 7 after treatment, asexual parasite densities had declined 249-fold and 1,240-fold, respectively, compared to pre-treatment. Gametocytes persisted at densities only 1.2-fold and 1.8-fold lower than pre-treatment at days 2 and 7, respectively (Fig. 1A-B, Supplementary Table 4). The median MOI of these blood stage parasites, consisting mainly of gametocytes (> 97%), was 2 (IQR 1–3) and 1 (IQR 1–2) at days 2 and 7 after treatment, respectively. Post-treatment clonality in paired infected mosquito midguts was lower, with a median MOI of 1 (IQR 1–2) at both timepoints. The clonality in blood stage parasites further declined to 33.3% (9/27) multiclonal human infections and 26.3% (5/19) multiclonal infected midguts at day 14 after treatment initiation, with a median MOI of 1 (IQR 1–2) in human samples and 1 (IQR 1–1) in infected midguts (Supplementary Figs. 4 and 5, Supplementary Table 6). No substantial variations were observed in gametocyte densities or gametocyte fractions (gametocyte density as a percentage of total parasite density) between monoclonal and polyclonal infections during study visits. Similarly, there were no significant differences in mosquito infection rates or oocyst densities in monoclonal and polyclonal infections (Supplementary Table 7).

### Transmission and persistence of minority clones

**Fig. 3:**
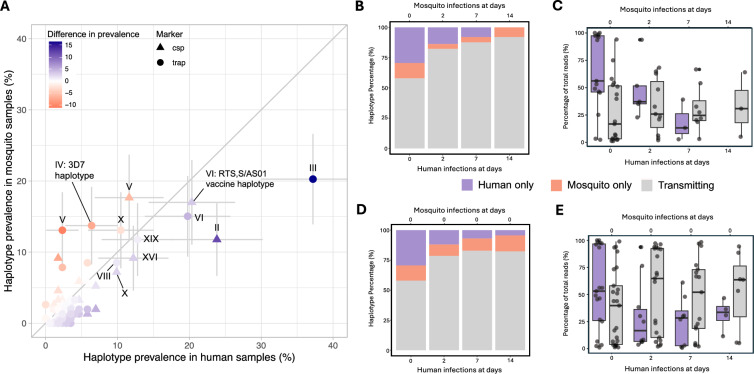
Molecular comparison of parasite clones in human infections and mosquito midguts post-feeding. **A** Differential prevalence of the *Pfcsp* and *Pftrap* haplotypes in the human and mosquito samples. Roman numbers represent the haplotypes in Fig. 2 haplotype networks. Error bars (representing 95% CI) and annotations are presented for haplotypes with at least 10% prevalence in either population. **B** Percentage of transmitting haplotypes and species-exclusive haplotypes when comparing human and mosquito samples at each timepoint (e.g. comparing haplotypes from day 2 human blood samples with haplotypes from infected midguts of mosquitoes that fed on the same day 2 blood material). **C** Percentage of transmitting haplotypes and species-exclusive haplotypes when comparing baseline mosquito samples to human samples at each timepoint (e.g. comparing haplotypes from day 2 human blood samples with haplotypes from infected midguts of mosquitoes that fed on day 0 blood material). **D** The percentage of total reads that transmitting and non-transmitting haplotypes encompass at each timepoint when comparing human and mosquito samples at each timepoint and **E** when comparing baseline mosquito samples to the human blood samples at each timepoint

Considering clones found in humans and cognate mosquitoes as whole populations, certain haplotypes were found to be more prevalent in human samples compared to mosquito samples and vice versa (Fig. 3A, Supplementary Fig. 6). To assess transmission between hosts and cognate mosquitoes in more depth, only pairwise human-mosquito groups were considered; human blood samples that did not have any cognate and successfully genotyped infected midguts were excluded and both COI markers were considered together, with percentages representing an average of both markers. Of all clones present in any species at baseline or on day 2/7, 30.1% was found to be present in baseline human infections while being absent in post-treatment (day 2/7) human infections and absent in mosquito infections (i.e. putative non-gametocyte-producers), 13.4% to be present in post-treatment (day 2/7) human infections, while absent in mosquito infections (i.e. putative gametocyte-producers, non-transmitting), and 56.5% to be present in mosquito infections (i.e. gametocyte-producers, transmitting). At baseline, prior to treatment initiation, 66.8% of haplotypes detected in participants transmitted to mosquitoes. A total of 82.3% of haplotypes detected in mosquitoes that had fed on baseline blood samples were observed in cognate human samples, while 29% of all haplotypes were found in human samples only and 12.6% of all haplotypes in mosquitoes only. At days 2 and 7, when > 99% of asexual parasites were removed but gametocytes persisted, the percentage of haplotypes detected in participants that transmitted to mosquitoes increased to 83.6% and 91.7%, respectively. The percentage of haplotypes observed in mosquitoes that were detected in cognate human samples increased as well to 95.3% and 95.4% at days 2 and 7, respectively. Of all haplotypes found in either species, the percentage of non-transmitting haplotypes, i.e. haplotypes that were exclusively detected in human samples decreased to 15.8% (day 2), 7.9% (day 7) and 0% (day 14), while the percentage of haplotypes that were only ever observed in mosquitoes decreased from 12.6% at baseline to 3.9% at day 2, and then increased again to 4.3% (day 7) and 7.8% (day 14) (Fig. 3B).

Transmitting haplotypes were often a minority clone at baseline, while they represented a higher percentage of sequencing reads at later timepoints (Fig. 3C), reflecting the rapid decline of asexual densities after treatment start, and the persistence of gametocytes post-treatment. With the aim of investigating whether the persisting clones are transmitted prior to treatment, the identity of clones in baseline infected mosquitoes were compared to human samples at all timepoints. This showed that the haplotypes that are transmitted at baseline match most closely with day 7 and 14 human samples (Fig. 3D). These matching haplotypes were often present as majority clones in the human blood samples at these timepoints (Fig. 3E). Transmission odds per haplotype showed differences in the likelihood of transmission for each haplotype, indicating that some haplotypes are more likely to transmit than others, at baseline and 48 h post-treatment (Supplementary Fig. 7).

### Differential transmission of drug resistance molecular markers

At day 2 after treatment, when the clones still present are majority gametocyte-producing, 49/50 (98%) human samples and 73/87 (83.9%) selected cognate mosquito samples were successfully assessed for the presence of anti-malarial drug resistance molecular markers with a median coverage of 990–6364 reads (Supplementary Table 5). A total of 610 amplicons were analysed across *Pfcrt*, *Pfmdr1*, *Pfdhfr*, *Pfdhps* and *Pfkelch13* genes.

**Fig. 4:**
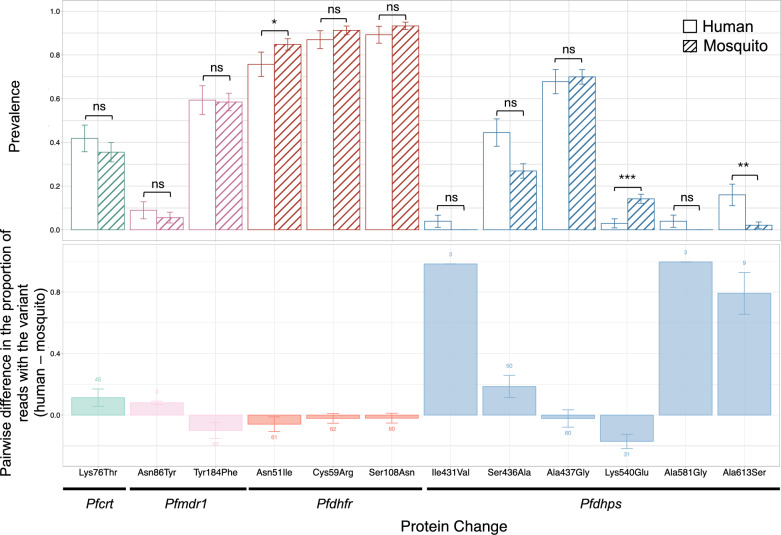
Molecular markers of drug resistance in human and mosquito samples. Prevalence of known single nucleotide polymorphisms linked to drug resistance in both species (upper panel). Pairwise comparison of human sample and cognate infected mosquito midgut, showing the difference in frequency of drug resistance markers between both (lower panel). ns = not significant, * = p < 0.05, ** = p < 0.01, *** = p < 0.001

As most infections were polyclonal, we assessed the frequency of the molecular markers in each sample, corresponding to the proportion of sequencing reads that contained the mutation. The prevalence of certain drug resistance polymorphisms was significantly different in blood stage and mosquito stage populations, with Asn51Ile in *Pfdhfr* and Lys540Glu in *Pfdhps* being significantly higher in mosquitoes (75.73% and 2.96% in blood stage parasites and 84.83% and 14.19% in mosquito midguts, p = 0.025 and p < 0.0001, respectively) and Ala613Ser in *Pfdhps* higher in blood stage parasites (15.99% in blood stage parasites and 2.13% in mosquito midguts, p = 0.0057, Fig. 4, Supplementary Table 8). In a pairwise comparison of cognate human blood and mosquito samples, the mean difference in frequency showed similarly that Asn51Ile and Lys540Glu may have a transmission advantage, while Ala613Ser shows a transmission disadvantage. One missense mutation in the propeller region of PfK13 was found (Val494Phe) in one infected midgut, however, this polymorphism has not been linked to partial artemisinin resistance (Supplementary Table 8).

## Discussion

In this study, amplicon sequencing was used to determine complexity of infection and prevalence of molecular markers of drug resistance, with the aim of investigating pre- and post-treatment parasite dynamics and genetic characteristics in blood stage parasites and oocysts from matched mosquito midguts. The results highlight the important role of gametocyte complexity and infectivity in creating the extensive diversity of *P. falciparum* genotypes found in infected individuals, in this area of seasonal transmission.

In baseline human blood stage parasites, 88.4% (38/43) of infections were polyclonal, and frequencies of drug resistance molecular markers were consistent with previous reports from this area [22, 23, 33]. As mosquitoes were dissected 7 days after feeding, any residual parasite material from the blood meal would have been eliminated [34]. Prior to treatment, minority clones were preferentially transmitted to mosquitoes. This may be due to majority clones representing new infections that consist predominantly of asexual parasites, which are present at higher densities than the’minority’ gametocytes [35]. New infections may not have had sufficient time to produce gametocytes, while gametocytes may represent a greater proportion of the parasite biomass in older infections. Alternatively, this observation aligns with the hypothesis of Berry et al*.* [12] reporting a selective advantage of minority clones in the vector; one biological hypothesis for this preferential transmission would be the maintenance of genetic diversity in the parasite population, which may be important with respect to the spread of drug resistance polymorphisms [12, 36]. These findings of clones detectable exclusively in the mosquito confirm previous reports that gametocytes present in blood stage infections at undetectable densities, potentially due to selective amplification of asexual parasites, are infectious for the mosquito vector [1, 11, 12]. In contrast with a study observing higher genetic diversity in mosquitoes [8], a higher clonality in human blood samples compared to infected mosquito midguts was found in this study. This could be attributed to the naturally infected mosquitoes that were investigated in that study, as compared to experimentally infected mosquitoes that were investigated here. Naturally infected mosquitoes can have taken multiple feeds on infected hosts, allowing parasite strains to accumulate in the mosquito abdomen. Comparing pre- and post-ACT, clones that persisted in the blood generally shared identity with clones present in cognate mosquitoes, as expected based on prior reports showing the persistence of gametocytes after most standard treatments, with the exception of artemether-lumefantrine [13–15, 37, 38]. These gametocyte-producing clones can persist until day 28 in some individuals, even after re-treatment with an ACT at day 21, supporting the addition of a single-low dose of primaquine to accelerate gametocyte clearance and preventing transmission.

A transmission advantage caused by a certain genetic variation can be the result of a higher gametocyte production, a higher gametocyte longevity or a more efficient fertilisation. The latter is evidenced by cases of gametocyte-producing clones after treatment that fail to infect mosquitoes, confirming that factors other than gametocyte density play a role in establishing oocyst development. Advantages in human-to-mosquito transmission have previously been observed in chloroquine-resistant parasites strains [18], and more recently in artemisinin-resistant malaria parasites under artemisinin drug pressure [39, 40]. Sulfadoxine-pyrimethamine resistant isolates were found to produce more gametocytes, but with unknown effect on transmission [19, 20]. In this study, nanopore sequencing was used to identify molecular markers of drug resistance and assess their prevalence in blood stage parasites at 48 h after ACT initiation and in matched infected mosquito midguts. The polymorphisms Asn51Ile in *Pfdhfr,* conferring pyrimethamine resistance*,* and Lys540Glu in *Pfdhps,* conferring sulfadoxine resistance, appeared to have a transmission advantage. In addition, Ala613Ser in *Pfdhps* was significantly more prevalent in human blood samples than in infected midguts and could, therefore, be associated with a transmission disadvantage. Notably, the Lys540Glu and Ala613Ser variants in *Pfdhps* were never observed together in the same infection. These mutations may be rendering the parasite intrinsically more or less infectious, in the absence of sulfadoxine-pyrimethamine drug pressure. In vitro studies introducing these polymorphisms with gene editing are needed to investigate a causal relationship between the polymorphisms and a change in parasite transmissibility. Additionally, a small but statistically insignificant transmission disadvantage of drug resistance marker Lys76Thr in *Pfcrt*, linked to chloroquine resistance, was observed, which is consistent with a previous report from Zambia showing preferential transmission of the wild-type (Lys76) form of *Pfcrt* compared to the mutant 76Thr [41], while another study found similar frequencies of wild-type and mutant *Pfcrt* alleles in gametocytes and sporozoite samples [12]. Monitoring the relative infectivity of drug-resistant mutations, including *Pfkelch13* mutations in areas with artemisinin partial resistance, may help model the spread of resistance.

This study had several limitations. Firstly, many infected midguts failed to amplify parasite DNA by PCR in the complexity of infection assay. This could be due to the midgut storage conditions and sensitivity of the assay. Consequently, the data presented here may have incurred a density bias, if midguts with a higher number of oocysts were more likely to be amplified compared to those with a lower number of oocysts. Although the median number of oocysts in infected midguts that amplified was identical to the midguts that did not amplify (median oocyst density of 1), the 75th percentile was higher in the amplified group (IQR 1–4 vs. IQR 1–2), indicating that a certain degree of density bias may have occurred. In addition, the mosquitoes used in the feeding assays were insectary-reared and, therefore, may be genetically different and have less overall genetic diversity than the natural population of mosquitoes in Mali. As the insectary-reared mosquitoes likely harbour low genetic diversity in midgut receptors for parasite invasion and development, this could affect transmission results [3]. Furthermore, the drug resistance polymorphisms assessed may not be selected for by the anti-malarial treatments administered in this study, and no artemisinin partial resistance has been reported in Mali to date. Complexity of infection markers and the drug resistance markers only represent a very small portion of the genome and it is not currently possible to “phase” this genotypic data. Therefore, the complexity of infection data cannot be linked to the observed frequencies of drug resistance markers for a specific sample. For example, in a blood sample containing four parasite clones, it is unfeasible to determine which clones possess drug resistance polymorphisms and which do not. There is a need for bioinformatic tools that enable phasing of this genetic variation in polyclonal infections, as this would offer valuable insights into whether drug resistance is present in the minority or majority parasite clone and whether transmitting or non-transmitting gametocyte-producing clones are drug resistant. Parasite sexual recombination takes place inside in the mosquito, leading to the creation of new parasite haplotypes; however, as another consequence of the short segments that were assessed in this study, it is highly unlikely that meiotic recombination occurred in the sequenced portion of the genome [42]. This suggests that the analysis presented here may have underestimated the parasite genomic diversity in infected mosquitoes. Finally, a study with a larger sample size and a wider range of infection densities is needed to draw epidemiological conclusions about which age groups have a higher multiplicity of infection and infectivity.

Overall, preferential transmission of minority parasite clones and putatively gametocyte-producing clones with SP resistance-conferring polymorphisms was observed in our study. These findings underscore the intricate nature of parasite-parasite and host-parasite interactions in their natural environments. They further stress the need for both fundamental and field studies to assess the importance of genetic and biological parasite and vector characteristics in driving parasite transmission. Molecular characterization of transmission could prove beneficial in the fight against drug resistance.

## Supplementary Information

Supplementary Material 1

## Data Availability

The sequence data presented in this study can be found in the European Nucleotide Archives (ENA, Project accession PRJEB73503).
