# Supplementary material for: Preferential transmission of minority and drug-resistant clones in polyclonal infections in Mali
Source: Malar J. 2025 Apr 5;24:111. doi: 10.1186/s12936-025-05298-6 (PMC11972506; doi:10.1186/s12936-025-05298-6)
Supplement: Supplementary file 1 — Supplementary Material 1 [file 12936_2025_5298_MOESM1_ESM.docx]

**SUPPLEMENTARY APPENDIX**

[Supplementary Figure 1. Diagram of sample selection and genotyping 2](#_Toc176260055)

[Supplementary Table 1. Primer sequences, barcodes and PCR conditions for complexity of infection markers 3](#_Toc176260056)

[Supplementary Table 2. Primer sequences and PCR conditions for drug resistance markers 4](#_Toc176260057)

[Supplementary Figure 2. Detection of minority clones 5](#_Toc176260058)

[Supplementary Table 3. Parasite prevalence and densities 6](#_Toc176260059)

[Supplementary Table 4. Infectivity to mosquitoes in infectious individuals 7](#_Toc176260060)

[Supplementary Table 5. Coverage across markers 8](#_Toc176260061)

[Supplementary Figure 3. Correlation between replicates and markers 9](#_Toc176260062)

[Supplementary Figure 4. Percentage of polyclonal infections 10](#_Toc176260063)

[Supplementary Figure 5. Median MOI at all timepoints in both species 11](#_Toc176260064)

[Supplementary Table 6. Percentage of polyclonal infections and median MOI at all timepoints 12](#_Toc176260065)

[Supplementary Table 7. Gametocyte densities, gametocyte fraction, mosquito infection rate and oocyst densities in monoclonal versus multiclonal infections 13](#_Toc176260066)

[P-values were calculated by Wilcoxon rank sum test. ref= reference, nc = not calculable, no observations/no observations over the threshold density for analysis 13](#_Toc176260067)

[Supplementary Figure 6. Haplotype count in human and mosquito hosts 14](#_Toc176260068)

[Supplementary Figure 7. Odds of transmission per haplotype 15](#_Toc176260069)

[Supplementary Table 8. Non-synonymous single nucleotide polymorphisms in genes associated with drug resistance. 16](#_Toc176260070)

## Supplementary Figure 1. Diagram of sample selection and genotyping

Fifty study participants were treated with dihydroartemisinin-piperaquine or pyronaridine artesunate. Blood samples were collected from participants prior to treatment (d0), during treatment (d2) and days 7, 14, 21 and 28 after treatment initiation, for infectivity assessment with direct membrane feeding assays, as well as for the quantification of asexual parasite and gametocyte densities. All individuals were retreated with dihydroartemisinin-piperaquine at day 21 to prevent re-infection. If the infectivity assays infected any number of mosquitoes at a certain timepoint for a certain individual, a maximum of three infected mosquitoes were selected at random per study participant per timepoint for complexity of infection genotyping (all timepoints) and drug resistance genotyping (day 2 only).

## Supplementary Table 1. Primer sequences, barcodes and PCR conditions for complexity of infection markers

| **Primer name** | **Sequence** |
| --- | --- |
| csp F | TTAAGGAACAAGAAGGATAATACCA |
| csp R | AAATGACCCAAACCGAAATG |
| trap F | TCCAGCACATGCGAGTAAAG |
| trap R | AAACCCGAAAATAAGCACGA |

**Forward barcodes**

| **BC1** | CTATCACG | **BC6** | CATCTAAC | **BC21** | AACCAAGG | **BC26** | CAACCATG |
| --- | --- | --- | --- | --- | --- | --- | --- |
| **BC2** | TCCAGTGT | **BC7** | TACAGATC | **BC22** | AAGGTACG | **BC27** | CTTCGAAG |
| **BC3** | GATCAGTA | **BC8** | CGTCTTGT | **BC23** | ACCTACCT | **BC28** | CAGAAGTG |
| **BC4** | AGTGTCGG | **BC9** | TATGATCA | **BC24** | ACTGGACT | **BC29** | CAGTGACT |
| **BC5** | GTAGCGCT | **BC10** | GGTAGCTT | **BC25** | ATATGCCG | **BC30** | CATGTGGT |

**Reverse barcodes**

| **BC11** | ATGGCTAG | **BC16** | GGGACTAC | **BC31** | CGTAGGAA | **BC36** | TCACTCTG |
| --- | --- | --- | --- | --- | --- | --- | --- |
| **BC12** | GACTTGGT | **BC17** | ACGTACTG | **BC32** | GACATCTG | **BC37** | TCTCCAGT |
| **BC13** | TCGATCAC | **BC18** | TGATTGCC | **BC33** | GCAATAGG | **BC38** | TGGTTCCT |
| **BC14** | ACACGTCA | **BC19** | AACTCTAC | **BC34** | GACACTGT | **BC39** | TGTGACTG |
| **BC15** | CAATGTGC | **BC20** | TGACTCAA | **BC35** | GTGAGTCT | **BC40** | GTCTACAG |

**PCR reaction**

| **Component** | **Volume per reaction (μL)** |
| --- | --- |
| Q5 buffer | 5 |
| Q5 enzyme | 0.25 |
| dNTPs | 0.5 |
| ddH_2_0 | 9.25 |
| Primers (at 10 μM) | 1.25 CSP F, 1.25 CSP R  1.25 TRAP F, 1.25 TRAP R |
| Template | 5 |
| **Total** | 25 |

| **PCR programme** | | |
| --- | --- | --- |
| **Step** | **Temperature** | **Duration** |
| Initial denature | 98 | 30 sec |
| For 30 cycles: |  |  |
| Denature | 98 | 10 sec |
| Annealing | 58 | 45 sec |
| Extending | 72 | 45 sec |
| Final extension | 68 | 2 min |

## Supplementary Table 2. Primer sequences and PCR conditions for drug resistance markers

| **Primer name** | **Sequence** | **PCR reaction** |
| --- | --- | --- |
| crt F | TGTCTTGGTAAATGTGCTCA | 1 |
| crt R | AGTTGTGAGTTTCGGATGTT | 1 |
| dhfr F | GTTTTCGATATTTATGCCATATGTG | 1 |
| dhfr R | TGATAAACAACGGAACCTCC | 1 |
| dhps F | TTTGTTGAACCTAAACGTGC | 2 |
| dhps R | AACATTTTGATCATTCATGCAAT | 2 |
| mdr1 F | TGTGTTTGGTGTAATATTAAAGAACA | 1 |
| mdr1 R | ACATAAAGTCAAACGTGCATTT | 1 |
| kelch13 F | AAGCCTTGTTGAAAGAAGCA | 2 |
| kelch13 R | GGGAACTAATAAAGATGGGCC | 2 |

**PCR reaction 1**

| **PCR programme** | | |
| --- | --- | --- |
| **Step** | **Temperature** | **Duration** |
| Initial denature | 98 | 30 sec |
| For 35 cycles: |  |  |
| Denature | 98 | 10 sec |
| Annealing | 60 | 35 sec |
| Extending | 72 | 45 sec |
| Final extension | 68 | 2 min |
| Store | 10 | Forever |

| **Component** | **Volume per reaction (μL)** |
| --- | --- |
| Q5 buffer | 10 |
| Q5 enzyme | 0.5 |
| dNTPs | 2.25 |
| ddH_2_0 | 30.05 |
| Primers (at 50 μM) | 0.25 CRT F, 0.25 CRT R  0.25 mdr1 F, 0.25 mdr1 R  0.6 dhfr F, 0.6 dhfr R |
| Template | 5 |
| **Total** | 50 |

**PCR reaction 2**

| **Component** | **Volume per reaction (μL)** |
| --- | --- |
| Q5 buffer | 10 |
| Q5 enzyme | 0.5 |
| dNTPs | 2.25 |
| ddH_2_0 | 27.45 |
| Primers (at 50 μM) | 1.2 dhps F, 1.2 dhps R  1.2 K13 F, 1.2 K13 R |
| Template | 5 |
| **Total** | 25 |

## Supplementary Figure 2. Detection of minority clones

Stacked bar chart displaying the percentage of reads attributed to each haplotype. All values shown are the average of both the replicates that were conducted for each dilution of 3D7/HB3.

## Supplementary Table 3. Parasite prevalence and densities

| Day of follow-up | Treatment arm | Asexual parasites | | Total gametocytes | | Ratio median asexual parasites / gametocytes density (%) |
| --- | --- | --- | --- | --- | --- | --- |
|  |  | Prevalence  % (n/N) | Density  Median parasites/μL (IQR) | Prevalence  % (n/N) | Density  Median parasites/μL (IQR) |  |
| Day 0 | *Overall* | 91.67% (44/48) | 397 (54.2-1932) | 100% (48/48) | 77.3 (37.1-124) | 513.58 |
|  | DHA-PPQ | 100% (25/25) | 673 (156-2342) | 100% (25/25) | 74.5 (37.6-126) | 903.36 |
|  | PY-AS | 82.61% (19/23) | 305 (31.2-1572) | 100% (23/23) | 83.2 (35.9-122) | 366.59 |
| Day 2 | *Overall* | 41.67% (20/48) | 1.59 (0.55-2.5) | 100% (48/48) | 62.6 (30.2-117) | 2.54 |
|  | DHA-PPQ | 48% (12/25) | 1.52 (0.45-2.21) | 100% (25/25) | 67.9 (33.9-119) | 2.24 |
|  | PY-AS | 34.78% (8/23) | 1.78 (0.66-3.19) | 100% (23/23) | 57.1 (28-99.5) | 3.12 |
| Day 7 | *Overall* | 28.57% (14/49) | 0.32 (0.22-0.64) | 100% (49/49) | 41.6 (17.0-68.2) | 0.77 |
|  | DHA-PPQ | 36% (9/25) | 0.24 (0.14-0.51) | 100% (25/25) | 41.6 (16.1-68.2) | 0.58 |
|  | PY-AS | 20.83% (5/24) | 0.55 (0.27-0.68) | 100% (24/24) | 41.4 (18.0-54.1) | 1.33 |
| Day 14 | *Overall* | 25.53% (12/47) | 0.47 (0.29-1.2) | 97.87% (46/47) | 21 (10.2-37.1) | 2.24 |
|  | DHA-PPQ | 30.43% (7/23) | 0.48 (0.29-1.14) | 100% (23/23) | 19.2 (8.49-46.5) | 2.50 |
|  | PY-AS | 20.83% (5/24) | 0.36 (0.35-1.01) | 95.83% (23/24) | 23.8 (10.2-33.5) | 1.51 |
| Day 21 | *Overall* | 16.67% (7/42) | 0.62 (0.35-1.08) | 95.24% (40/42) | 5.25 (2.62-13.6) | 11.81 |
|  | DHA-PPQ | 14.29% (3/21) | 0.57 (0.35-0.6) | 95.24% (20/21) | 5.94 (2.60-18.5) | 9.60 |
|  | PY-AS | 19.05% (4/21) | 1.08 (0.59-3.42) | 95.24% (20/21) | 4.51 (2.85-8.84) | 23.95 |
| Day 28 | *Overall* | 9.3% (4/43) | 0.08 (0.05-1) | 90.7% (39/43) | 0.83 (0.14-2.69) | 9.64 |
|  | DHA-PPQ | 17.39% (4/23) | 0.08 (0.05- 0.99) | 91.3% (21/23) | 1.44 (0.17-4.06) | 5.56 |
|  | PY-AS | 0% (0/20) | . | 90% (18/20) | 0.76 (0.14-1.51) | . |

DHA-PPQ=dihydroartemisinin-piperaquine, PY-AS=pyronaridine-artesunate

## Supplementary Table 4. Infectivity to mosquitoes in infectious individuals

| Day of follow-up | Treatment arm | Infectious individuals % (n/N) | Mosquito infection rate^*^  Median % (IQR) | Oocyst density^**^  Median (IQR) |
| --- | --- | --- | --- | --- |
| Day 0 | *Overall* | 33/50 | 14.9 (3.51-31.4) | 1 (1-2) |
|  | DHA-PPQ | 16/25 | 23.6 (8.64-35.6) | 1 (1-2) |
|  | PY-AS | 17/25 | 6.15 (2.99-24) | 1 (1-2) |
| Day 2 | *Overall* | 35/50 | 10 (3.61-25) | 1 (1-2) |
|  | DHA-PPQ | 19/25 | 11.5 (3.06-25.8) | 1 (1-2) |
|  | PY-AS | 16/25 | 9.49 (4.26-23.6) | 1 (1-1.5) |
| Day 7 | *Overall* | 31/50 | 5.33 (2.7-27.8) | 1 (1-1) |
|  | DHA-PPQ | 17/25 | 7.69 (3.03-27) | 1 (1-1) |
|  | PY-AS | 14/25 | 3.85 (2.1-33.3) | 1 (1-1) |
| Day 14 | *Overall* | 15/49 | 7.69 (3.85-27.4) | 1 (1-1.5) |
|  | DHA-PPQ | 10/24 | 6.65 (2.98-28.5) | 1 (1-1.38) |
|  | PY-AS | 5/25 | 17.2 (8.62-22.7) | 1 (1-1.5) |
| Day 21 | *Overall* | 7/23 | 3.03 (2.12-11.2) | 1 (1-1) |
|  | DHA-PPQ | 5/15 | 3.03 (2.7-8.2) | 1 (1-1) |
|  | PY-AS | 2/8 | 10.5 (6-15) | 1 (1-1) |
| Day 28 | *Overall* | 2/18 | 2.22 (1.81-2.62) | 1 (1-1) |
|  | DHA-PPQ | 1/12 | 1.41 (1.41-1.41) | 1 (1-1) |
|  | PY-AS | 1/6 | 3.03 (3.03-3.03) | 1 (1-1) |

^*^In infectious individuals

^**^In infectious individuals, median number of oocysts in infected mosquitoes

DHA-PPQ=dihydroartemisinin-piperaquine, PY-AS=pyronaridine-artesunate

## Supplementary Table 5. Coverage across markers

| Marker | Genome positions | Median coverage [IQR] |
| --- | --- | --- |
| *Pfcsp* | Pf3D7_03_v3: 221,352-221,640 | 1002 [287-3360] |
| *Pftrap* | Pf3D7_13_v3: 1,465,059-1,465,378 | 944 [258-3244] |
| *Pfcrt* | Pf3D7_07_v3: 403,536-403,673 | 6364 [772-23033] |
| *Pfmdr1* | Pf3D7_05_v3: 958,143-958,459 | 2812 [70.5-9948] |
| *Pfdhfr* | Pf3D7_04_v3: 748,134-748,579 | 2790 [58-8391] |
| *Pfdhps* | Pf3D7_08_v3: 922,591-923,189 | 3528 [47-11726] |
| *Pfk13* | Pf3D7_13_v3: 1,724,874-1,725,701 | 990 [21-6071] |

## Supplementary Figure 3. Correlation between replicates and markers

Spearman correlation coefficient was calculated to assess the correlation between markers and replicates. MOI = Multiplicity of infection.

## Supplementary Figure 4. Percentage of polyclonal infections

## Supplementary Figure 5. Median MOI at all timepoints in both species

MOI = multiplication of infection.

## Supplementary Table 6. Percentage of polyclonal infections and median MOI at all timepoints

| Day of follow-up | Proportion of multiclonal infections in blood stage parasites  % (n/N) | Proportion of multiclonal infections in infected midguts  % (n/N) | Median MOI blood stage parasites (IQR) | Median MOI infected mosquito midguts (IQR) |
| --- | --- | --- | --- | --- |
| Day 0 | 80.37% (38/43) | 36.54% (19/52) | 3 (2-5) | 1 (1-2) |
| Day 2 | 62.5% (25/40) | 42.11% (16/38) | 3 (1-3) | 1 (1-2) |
| Day 7 | 37.2% (19/43) | 40% (14/35) | 1 (1-2) | 1 (1-2) |
| Day 14 | 33.33% (9/27) | 26.32% (5/19) | 1 (1-2) | 1 (1-2) |
| Day 21 | 54.55% (6/11) | 33.34% (1/3) | 2 (1-2) | 1 (1-1.5) |
| Day 28 | 20% (1/5) | 0% (0/2) | 1 (1-1) | 1 (1-1) |

Supplementary Table 7. Gametocyte densities, gametocyte fraction, mosquito infection rate and oocyst densities in monoclonal versus multiclonal infections

| Day of follow-up | MOI | Gametocyte density | | Gametocyte fraction (gametocytes/total parasites) | | Mosquito infection rate | | Oocyst density | |
| --- | --- | --- | --- | --- | --- | --- | --- | --- | --- |
|  |  | Median parasites/μL (IQR) | p-value | Median % (IQR) | p-value | Median % (IQR) | p-value | Median % (IQR) | p-value |
| Day 0 | 1 | 80.2 (62.4-145) | ref | 77.6 (0.23-82.3) | ref | 24 (6.15-31.4) | ref | 2 (1-4) | ref |
|  | >1 | 80.3 (37.8-123) | 0.698 | 20.4 (5.33-58.2) | 0.756 | 2.92 (0-21.1) | 0.105 | 3 (1-6.5) | 0.251 |
| Day 2 | 1 | 66.0 (32.2-102) | ref | 100 (99.2-100) | ref | 9.52 (1.75-19.7) | ref | 1 (1-3.75) | ref |
|  | >1 | 96.7 (40.2-157) | 0.199 | 100 (98.7-100) | 0.748 | 4.35 (1.56-21.9) | 0.888 | 2.5 (1-62.5) | 0.132 |
| Day 7 | 1 | 41.3 (17.0-49.8) | ref | 100 (99.6-100) | ref | 2.13 (0-4.41) | ref | 1 (1-1.75) | ref |
|  | >1 | 47.5 (34.4-105) | 0.0756 | 100 (99.8-100) | 0.989 | 5.33 (0.76-25.8) | 0.199 | 2 (1-17) | 0.0489 |
| Day 14 | 1 | 25.4 (11.8-43.7) | ref | 100 (100-100) | ref | 0 (0-6.65) | ref | 1 (1-2) | ref |
|  | >1 | 28.6 (14.9-48.3) | 0.555 | 100 (99.4-100) | 0.0434 | 0 (0-4.48) | 0.867 | 1.5 (1-2.75) | 0.421 |
| Day 21 | 1 | 41.5 (26.2-105) | ref | 100 (99.7-100) | ref | 1.54 (0-8.2) | ref | 1 (1-1) | ref |
|  | >1 | 28.3 (15.2-47.8) | 0.648 | 100 (100-100) | 0.486 | 0.76 (0-2.41) | 0.503 | 1.5 (1-2.75) | nc |
| Day 28 | 1 | 14.1 (6.63-23.0) | ref | 100 (100-100) | ref | 0 (0-0.35) | ref | 1 (1-1) | ref |
|  | >1 | 31.4 (31.4-31.4) | 0.289 | 100 (100-100) | nc | 3.03 (3.03-3.03) | 0.236 | nc | nc |

P-values were calculated by Wilcoxon rank sum test. ref= reference, nc = not calculable, no observations/no observations over the threshold density for analysis

Supplementary Figure 6. Haplotype count in human and mosquito hosts

## Supplementary Figure 7. Odds of transmission per haplotype

Haplotypes were only included if their transmissibility was assessed more than once, with the white number representing the occurrence count. Roman haplotype names correspond to the haplotypes in Figure 2 and are only presented in the figure if the odds of transmission > 0.1.

## Supplementary Table 8. Non-synonymous single nucleotide polymorphisms in genes associated with drug resistance.

| Gene | Gene ID | Nt change | AA change | Human blood *Pf* samples MAF  (%) n = 48 | Mosquito midgut *Pf* samples MAF (%) n = 73 | P value* |
| --- | --- | --- | --- | --- | --- | --- |
| *Pfcrt* | PF3D7_0709000 | 403625A>C | Lys76Thr | 41.81 | 35.49 | 1 |
| *Pfmdr1* | PF3D7_0523000 | 958145A>T | Asn86Tyr | 8.94 | 5.59 | 0.506 |
|  |  | 958440A>T | Tyr184Phe | 59.31 | 58.45 | 0.121 |
| *Pfdhfr* | PF3D7_0417200 | 748239A>T | Asn51Ile | 75.73 | 84.83 | 0.025 |
|  |  | 748262T>C | Cys59Arg | 86.97 | 91.24 | 0.384 |
|  |  | 748410G>A | Ser108Asn | 89.23 | 93.28 | 0.300 |
| *Pfdhps* | PF3D7_0810800 | 549666A>G | Ile431Val | 3.93 | 0 | 0.158 |
|  |  | 549681T>G | Ser436Ala | 44.46 | 26.91 | 0.852 |
|  |  | 549682C>T | Ser436Phe | 1.55 | 0 | nc |
|  |  | 549682C>A | Ser436Tyr | 0 | 0.83 | nc |
|  |  | 549685G>C | Ala437Gly | 67.76 | 69.95 | 0.178 |
|  |  | 549993A>G | Lys540Glu | 2.96 | 14.2 | <0.001 |
|  |  | 550087G>T | Arg571Met | 0 | 0.19 | nc |
|  |  | 550098G>T | Asp575Tyr | 0 | 0.43 | nc |
|  |  | 550117C>G | Ala581Gly | 3.95 | 0 | 0.155 |
|  |  | 550212G>T | Ala613Ser | 15.99 | 2.13 | 0.0057 |
| *PfK13* | PF3D7_1343700 | 1725518G>T | Val494Phe | 0 | 0.13 | nc |

MAF = Minor allele frequency; Nt = Nucleotide; AA = Amino Acid

*Comparing human blood *Pf* samples and mosquito midgut *Pf* samples
